# Empowering Men’s Health: Strategies to Enhance Health-Seeking Behaviour in Rural Limpopo, South Africa

**DOI:** 10.1177/11786329261472590

**Published:** 2026-07-31

**Authors:** Lazarros Chavalala, Lufuno Makhado, Rachel Tsakani Lebese

**Affiliations:** 1 Department of Public Health, Faculty of Health Sciences, 56868 University of Venda, Thohoyandou, South Africa

**Keywords:** enhance, health, health-seeking behaviour, men, strategies

## Abstract

**Introduction:**

Globally, men have reportedly experienced poor health outcomes associated with their poor health-seeking behaviour. Implementing various strategies could help improve men’s health-seeking behaviour and achieve better health outcomes among men. This study aimed to develop strategies to improve men’s health-seeking behaviour among men in Limpopo Province, South Africa.

**Methods:**

A mixed-method multi-phase approach was used. In phase 1, a systematic review was conducted on strategies used to improve men’s health-seeking behaviour to evaluate their effectiveness. In phase 2, quantitative and qualitative studies were conducted, and their findings were merged. In phase 3, Strengths, Weaknesses, Opportunities, and Threats (SWOT) analysis was applied to the merged findings to identify factors that are categorised as strengths, weaknesses, opportunities, and threats. The SWOT analysis outcomes were subjected to the Build, Overcome, Explore, and Minimise (BOEM) Model. The BOEM-derived actions were then integrated and used to formulate strategies.

**Results:**

A total of 14 strategies were developed and include recognition of the best employees, implementation of patient surveys, intensifying compliance with policies and guidelines, reduction of waiting time, regular staff training, employing male nurses and community health workers, community outreach campaigns, advocacy, introduction of male dedicated sections in existing health facilities, and use of technology.

**Conclusions:**

Applying the developed strategies could help improve men’s health-seeking behaviour and create awareness of the importance of utilising healthcare services among the male population. It could also address misconceptions and stigma surrounding the use of healthcare services in communities.

## Introduction

In many parts of the world, men engage less with health services than women, are less likely to access preventive services, and are more likely to drop out of care.^1^ They often delay seeking help for health-related problems and consider consulting when medical conditions become severe and potentially difficult to treat.^2^ Regardless of various reasons for delaying seeking care, there are consequences that men face, such as increased risk of HIV infection, mortality and infertility for those infected with STIs.^3^ With men observed to underutilise health services, various studies, including studies of^4-7^ were conducted to explore factors influencing men’s poor health-seeking behaviour. Research findings indicate that men find general health centres unwelcoming and unaccommodating, citing traditional masculine behaviour as a reason for delaying seeking care, use of alternative methods to address illness and financial constraints as explanations for not seeking healthcare.^4-7^ After the conducted studies identified contributing factors to men’s low utilisation of health services, strategies were recommended based on the factors found. Developed and recommended strategies included patient and/or physician communication strategy, community-based interventions, the introduction of male clinics, peer education, and men’s sheds.^8-12^ However, there were gaps identified that led to ineffectiveness in some of the developed strategies. This includes a lack of practical strategies to encourage men to use health services. Moreover, except for the introduction of male clinics and men’s sheds, the strategies were not gender responsive to men’s needs, including respect. Community-based interventions and peer education were short-term, lacked sustainability and relied on donor funding. Moreover, there were few studies conducted in Limpopo Province into factors contributing to men’s poor health-seeking behaviour and developed strategies to improve men’s health-seeking behaviour. Given the limited research on drivers of poor health-seeking behaviour among men in Limpopo Province, the authors considered it necessary to explore barriers contributing to poor health-seeking behaviour. The findings were intended to inform the development of context-specific strategies to address these barriers. The authors conducted this study guided by Andersen’s health service utilisation model^13^ because of its ability to explain health services utilisation. The model categorizes the factors influencing health service utilisation into three elements: (i) Predisposing factors refer mainly to the demographic traits of the participants in the study, and these characteristics might either enhance or reduce the chances that individuals will view treatment as a feasible option or alternative when confronted with illness; (ii) enabling factors are resources that boost the likelihood that individuals in need can access or use these services; (iii) need factors encompass both the perceived need and the actual requirement for health services.^14^ The model categorisation of factors informs analytical categorisation of barriers and facilitators influencing men’s use of health services. Subsequently, the constructs (predisposing, enabling and need factors) informed the identification of strengths, weaknesses, opportunities and threats within the SWOT analysis. The Build, Overcome, Explore and Minimise (BOEM) model leveraged SWOT analysis results to inform actions that build, overcome, explore, and minimise factors based on Andersen’s model constructs. As a result, the model has enabled the authors to explore and categorise factors contributing to men’s poor health-seeking behaviour in the context of using health services within the study area. Using information gathered on factors contributing to men’s poor health-seeking behaviour, the authors developed strategies to improve men’s health-seeking behaviour in Limpopo Province, South Africa. In this manuscript, the authors report on the processes followed in developing the proposed strategies to improve men’s health-seeking behaviour in Limpopo Province.

## Material and Methods

A multiphase convergent parallel mixed-methods design was used to achieve this study’s aim. Strategies development was done in three phases from January 2022 to October 2024, as shown in Table 1. Reporting of this study findings is in line with the Good Reporting of A Mixed Methods Study (GRAMMS) guideline developed by O’Cathain et al (2008).^15^ In Phase 1, a systematic review was conducted to identify strategies commonly used to improve men’s health-seeking behaviour and evaluate their effectiveness. Phase 2 was divided into three sub-phases: Phase 2A (quantitative studies), Phase 2B (qualitative studies), and Phase 2C (integration of findings). Empirical phase findings were presented in six drafted manuscripts, which were submitted for peer review in accredited journals. In Phase 2C, the findings from six papers (5 published and 1 under review) were combined. After completing Phase 2C, the researcher began developing strategies in Phase 3 using SWOT analysis and the Build, Overcome, Explore, and Minimise (BOEM) Model as shown in Table 1. To reduce duplication bias and overlap, overlapping findings from the papers were identified and counted only once during strategies development. Recurring themes were not treated as additional evidence and weight, but as confirmation of relevance and consistency.

**Table 1.** Summary of Study Phases

Study phase		Design/Method	Sample/Data source	Purpose	Contribution to strategy development
**Phase 1**		**Systematic review**	✓ published literature on interventions improving men’s health-seeking behaviour	✓ To identify existing interventions and evidence-based practices	✓ Informed identification of effective and ineffective intervention approaches
**Phase 2**	​	**Convergent Mixed Methods**	​	✓ To explore factors	✓ Identified contextual barriers and
	**Phase 2 A**	**Quantitative**	✓ Rural adult men	Measure men’s health awareness and determine their attitudes towards public health services	✓ Helped to establish an association between behavioural factors and HSB
	**Phase 2 B**	**Qualitative**	Rural adult men	Explore factors influencing poor health HSB	✓ Identified contextual factors influencing men’s HSB
			✓ Professional Nurses		
	**Phase 2 C**	**Parallel merging**	✓ Quantitative and Qualitative findings	Merge phase 2A and 2B findings	✓ Led to a better understanding of men’s HSB
**Phase 3**		**SWOT Analysis**	✓ Findings from mixed methods studies	✓ To categorise strengths, weaknesses, opportunities, and threats	✓ Contributed to identifying and categorising factors that influence the occurrence of research problems, and areas of improvement
		**BOEM Analysis**	✓ SWOT analysis findings	✓ To identify areas to Build, Overcome, Explore, and Minimise	✓ Guided formulation of context-specific intervention strategies
		**Strategy development**	✓ BOEM-derived actions	✓ Formulate strategies	✓ Produced evidence-informed and contextually relevant intervention strategies

### Phase 1: Systematic Review

**Figure 1.**
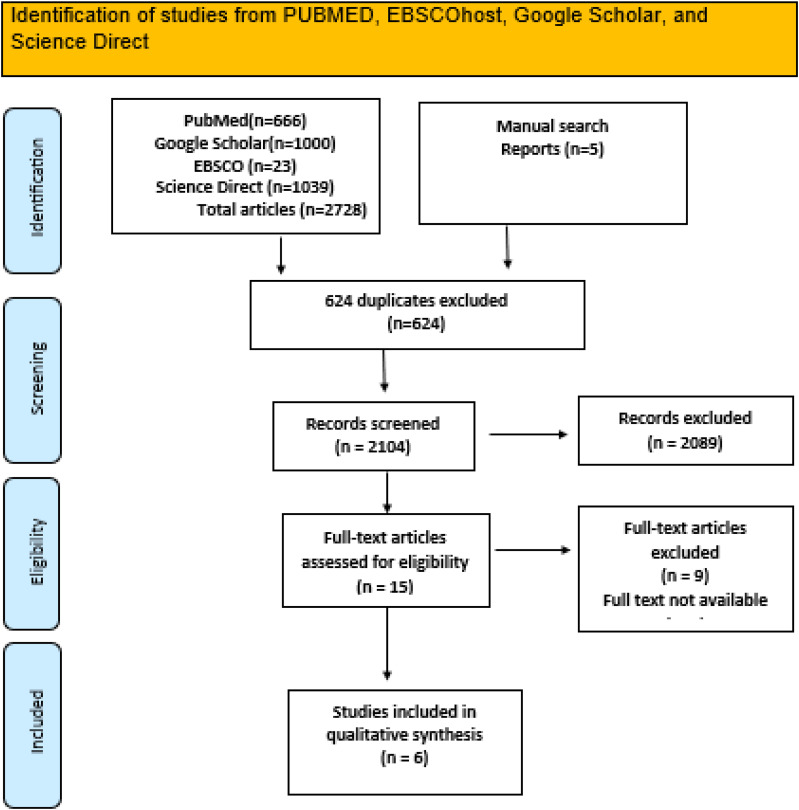
PRISMA flow chart

The systematic review presented in the Prisma flow diagram in Figure 1 identified strategies used in South Africa and other countries, and their gaps and evaluated their effectiveness in enhancing men’s health-seeking behaviour. It targeted studies that focused on strategies to promote men’s health-seeking behaviour, where men aged 18 and above were the recipients of the intervention. Only studies and reports published in peer-reviewed journals from January 2000 to December 2020 were considered, as it was a methodologically appropriate cutoff point at the time the review was conducted. PUBMED, EBSCOhost, Google Scholar, and ScienceDirect databases were used to search the literature. Grey literature was also considered. The keywords “health-seeking behaviour”, “strategies” and “men” were used to search for relevant literature. The authors screened the titles and abstracts independently to identify the relevant reports and articles, and those that met the inclusion criteria were used to collect data using a uniform data collection tool, as presented in Figure 1. Disagreements between the authors were resolved through dialogue. The suitability of the chosen studies was evaluated using the AMSTAR tool for evaluating the methodological quality of systematic reviews and the critical appraisal of included studies. It was clear from the findings of the paper titled “*Strategies to promote men’s health-seeking behaviour: A systematic review*” that there were not many previously used strategies put in place to improve men’s health-seeking behaviour, and the strategies had gaps and subsequently failed. This suggested that there was still a need to further explore factors affecting men’s health-seeking behaviour and develop evidence-based strategies that can be employed to improve men’s health. An empirical phase was conducted.

### Phase 2: Empirical Phase

Phase 2 was divided into three sub-phases, namely Phase 2A (quantitative studies), Phase 2B (qualitative studies), and Phase 2C (merging findings from papers). Both quantitative and qualitative studies were conducted concurrently, and their findings were presented in six drafted manuscripts. In Phase 2C, the findings from six manuscripts were merged.

#### Phase 2 A: Quantitative

Two studies titled “Evaluation of Health Awareness and Perceived Health Status among Men in Limpopo Province, South Africa” and “Men’s Attitudes Towards Public Health Services and Utilisation Practices in Limpopo Province, South Africa” were conducted, and their findings were presented in research papers and published. The studies employed cross-sectional descriptive designs. The Evaluation of Health Awareness and Perceived Health Status among Men in Limpopo Province, South Africa study included 387 randomly selected men aged 18 and older who resided within the study setting at the time of conducting the study. The study excluded males under 18 years old and those who could not consent to participate. The second quantitative study on Men’s Attitudes Towards Public Health Services and Utilisation Practices in Limpopo Province, South Africa, included 387 men randomly selected within the study setting. The study focused on men aged 18 and above and excluded those younger than 18 and those who did not consent to participate. Those who did not reside within the study setting were also excluded. The study sample sizes in both papers were determined using an online sample size calculator with a margin of error set at 5 per cent. Data for both papers were analysed independently using Statistical Package for Social Sciences (SPSS) version 29.0. Descriptive statistics and inferential statistics were used. The significance level was set at 0.05, and all tests were two-tailed. Ethical approval was granted by the University of Venda Research Ethics Committee with reference number FHS/21/PH/26/1215 on 15 December 2021, and community leaders gave permission to access men in these communities. All respondents had consented in writing to participate in the study after they were given full information about the study and how they were expected to participate. Major findings from the quantitative studies were men’s adequate knowledge about their health, a negative attitude towards public health services and irregular use of public healthcare facilities.

#### Phase 2 B: Qualitative

Three studies titled “*Men’s Views on Factors Contributing to Their Poor Health-seeking Behaviour in Limpopo Province, South Africa*”, “*Male Patients’ Experiences when Visiting Public Health Facilities in Limpopo Province, South Africa*”, and “*Professional Nurses’ Views and Experiences of Poor Health-seeking behaviour among Men in Limpopo Province*” were conducted. Findings of these studies were put into papers and have been published. The studies employed explorative designs. Purposive sampling was used independently in the studies based on sampling characteristics to select participants. For the study on Men’s Views on Factors Contributing to Their Poor Health-seeking Behaviour in Limpopo Province, South Africa, 21 men who had never visited health facilities to seek health services were purposively selected and excluded those who had sought health services.

For the study on “Male Patients’ Experiences when Visiting Public Health Facilities in Limpopo Province, South Africa”, 28 males who had visited public health facilities to use health services were purposively selected and excluded those who had never visited health facilities to seek health care. For the study on “Professional Nurses’ Views and Experiences of Poor Health-seeking behaviour among Men in Limpopo Province”, 14 professional nurses with experience of providing health services to males were purposively selected. Professional nurses without experience in providing males with health services were excluded. The sample size in these studies was determined by data saturation. Individual semi-structured interviews were held with individual participants for the papers “Men’s Views on Factors Contributing to Their Poor Health-seeking Behaviour in Limpopo Province, South Africa” and “Professional Nurses’ Views and Experiences of Poor Health-seeking behaviour among Men in Limpopo Province”. Focus group discussions were held with participants for the paper title “Male Patients’ Experiences when Visiting Public Health Facilities in Limpopo Province, South Africa” to collect data. Collected data were transcribed verbatim in Microsoft Word, transcripts were read in detail several times, and then codes and themes were manually developed. An independent coder was also sought for expert guidance and validation of the codes and themes that were generated. The University of Venda Research Ethics Committee granted ethical approval for these studies with reference number FHS/21/PH/26/1215 on 15 December 2021, and community leaders were permitted access to men in their communities. The Limpopo Provincial Department of Health granted permission to access health facilities and interview professional nurses. All participants gave consent to participate in writing by filling out consent forms after an explanation of the study’s details and how they were going to participate. Upon completion of the analysis of both qualitative and quantitative sub-phases, there was a need to merge findings, and the authors moved to phase 2C.

Two participants from “Men’s Views on Factors Contributing to Their Poor Health-seeking Behaviour in Limpopo Province, South Africa” said:

*“We as men or any men tell ourselves that we are strong, or our body is strong and is not like the body of women, that is what men tell themselves that coughing does not need one to run to the doctor or clinic, it is something that will pass*” Participant 1, male 34-year-old;

*“Us on our nature, even when we were growing up, we were trained to be strong, as you are strong, you will not run to the hospital when you are sick in most cases. Just like me, in a year, I can go once or twice”* Participant 8, male, 30 years old.

For a study on “Professional Nurses’ Views and Experiences of Poor Health-seeking behaviour among Men in Limpopo Province”, two participants said:

“*From the others that I’ve treated, you find that they’re very grateful after being serviced very well and treated with respect and dignity*” Clinician 7, female, 40-year-old;

“*You get people who are working in firms and didn’t come to the clinic because of work. They couldn’t get time off.”* Clinician 3, female, 35-year-old

#### Phase 2C: Merging Findings From Papers

Convergent parallel merging of findings from the six papers was done using a table where both qualitative and quantitative findings were presented side by side and compared with each other. This enabled the authors to identify areas where the findings complemented each other and where they differed. Paper two, titled “Evaluation of Health Awareness and Perceived Health Status Among Men in Limpopo Province, South Africa”, aimed to determine men’s health awareness about their health. The paper also aimed to measure the association between health awareness and men’s level of education. Paper three, titled “Men’s Attitudes Towards Public Health Services and Utilisation Practices in Limpopo Province, South Africa”, aimed at determining the attitudes of men towards public health services and utilisation practices. Paper four, titled “Men’s Views on Factors Contributing to their Poor Health-Seeking Behaviour in Limpopo Province, South Africa”, aimed to describe men’s views on the reasons for not visiting health facilities to access health services. This enabled the study to identify underlying factors associated with men’s poor health-seeking behaviour. Paper five, “Male patients’ experiences when visiting public health facilities in Limpopo Province, South Africa”, aimed at describing the experiences of Male patients during visits to public health facilities. This paper helped identify experiences contributing to men’s poor health-seeking behaviour. Moreover, Paper Six, titled “Professional Nurses’ Views and Experiences of Poor Health-Seeking Behaviour Among Men in Limpopo Province”, aimed at describing professional nurses’ views on factors contributing to men’s poor health-seeking behaviour. This paper also explored professional nurses’ experiences during treatment sessions with male patients.

### Phase 3: Strategy Development

In phase 3, SWOT analysis and the BOEM model were applied to merge findings from the papers. SWOT analysis was used to identify strengths, weaknesses, opportunities, and threats based on the findings. The BOEM model was used to develop actions that can be used to build on identified strengths, overcome weaknesses, explore identified opportunities, and minimise threats in men’s engagement with healthcare services. Integration of BOEM-derived actions through grouping of similar actions together with factors they addressed was done in tabular form, and strategies were formulated as an umbrella action of the proposed BOEM-derived actions.

### Application of SWOT Analysis

**Figure 2.**
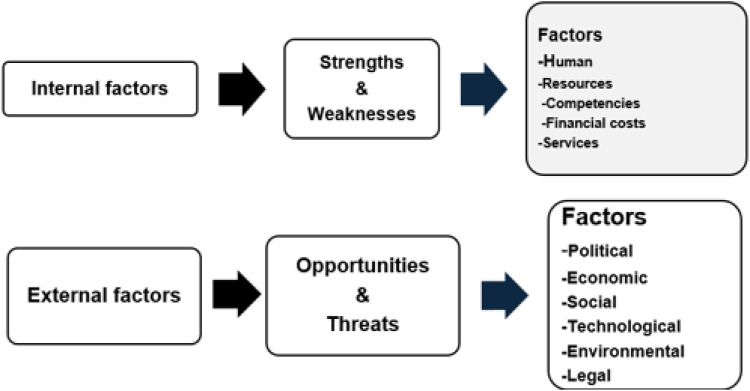
SWOT analysis category of factors contributing based on HRCFS and PESTEL

To systematically examine internal and external factors contributing to men’s poor health care utilisation. SWOT analysis was applied to enable authors to categorise factors into strengths, weaknesses, opportunities and threats, which were necessary to develop informed and evidence-based strategies that address men’s poor health-seeking behaviour. Strengths and weaknesses were classified as internal factors, as they relate to service delivery at the health facility level within the Department of Health that can be directly influenced, such as human resources, staff competencies, financial costs, and the organisation of services. In contrast, opportunities and threats were classified as external factors, as they arise from broader sociocultural, economic, and policy contexts beyond the direct control of the Department of Health. This distinction guided the placement of findings into the SWOT quadrants. For example, participants’ positive perceptions of respectful communication by healthcare workers were categorised as a strength, while concerns about long waiting times and the shortage of male clinicians were classified as weaknesses. On the other hand, existing guidelines guiding professional conduct and policies promoting primary healthcare access were identified as opportunities. Whereas sociocultural norms discouraging men from seeking care and low perceived need for preventive services were categorised as threats. The Political, Economic, Social, Technological, Environmental, and Legal (PESTEL) model was used to categorise factors considered as opportunities and threats in improving men’s engagement with health services (Figure 2).

### Application of the BOEM Model

Results of the SWOT analysis matrix, both on internal and external factors, were then subjected to the BOEM model. The BOEM model was chosen for its ability to recommend actions and leverage the SWOT analysis results in finding the solution to an identified problem. In applying the BOEM model, Strengths, Weaknesses, Opportunities, and threats were carefully examined. Actions to build on identified strengths within the public health system in addressing men’s poor health-seeking behaviour were developed. Actions to overcome weaknesses identified within the public health system were also developed. Opportunities to improve men’s health-seeking behaviour were identified and explored, and actions were developed to mitigate the threats hindering improvements in men’s health-seeking behaviour. Developed actions to strengthen identified strengths, address weaknesses within the public health system, together with actions to minimise identified threats contributing to men’s poor health-seeking behaviour and those exploring how identified opportunities could be used to overcome poor health-seeking behaviour were used to develop strategies.

### Integration of BOEM-Derived Actions

In this phase, BOEM-derived actions were integrated through grouping based on their similarities and the factors they were meant to address. Strategies incorporating BOEM-derived actions were then formulated to represent grouped BOEM-derived actions. Moreover, for BOEM-derived actions that were not related to other actions, strategies representing such actions were also formulated. BOEM-derived actions describe practical implementation activities to be executed, while formulated strategies present required actions.

## Results

### Merged Findings

The merged findings indicated that although men demonstrated adequate health knowledge, this did not translate into utilisation of health services, suggesting that behavioural, cultural, and systemic barriers outweigh knowledge in influencing health-seeking behaviour. Participants generally perceived their health status as good. However, despite this perception, they expressed dissatisfaction with public health services, citing poor service quality and long waiting times. Gender norms and expectations of masculinity significantly influenced health-seeking behaviour, with men expressing discomfort in female-dominated healthcare environments and reluctance to appear vulnerable. Men’s reliance on self-medication and traditional healing practices reflects competing healthcare pathways that may delay engagement with formal health services. Negative experiences within health facilities, including poor staff attitudes and breaches of confidentiality, contributed to men’s dissatisfaction and reluctance to seek care. Despite reported challenges, some clinicians were recognised for providing respectful communication and patient education, highlighting variability in service quality.

### SWOT Analysis Results

Regarding this study’s merged findings, Strengths, Weaknesses, Opportunities, and Threats were identified to foster an understanding of men’s poor health-seeking behaviour and factors. The findings are presented in Table 2.

**Table 2.** SWOT Analysis Outcomes

Strengths	Weaknesses
✓ Dedicated and Respectful Clinicians	✓ Long waiting period
✓ Patient Satisfaction	✓ Poor communication from Clinicians to patients
✓ Free, available, and accessible service	✓ Bad staff attitude
	✓ Delay in helping patients
	✓ Shortage of male clinicians
	✓ Confidentiality breach by health workers
	✓ Accessibility to health facilities
	✓ Poor monitoring of the implementation of the South African National Integrated Men’s Health Strategy 2020-2025

Opportunities	Threats
✓ Availability of social media platforms	✓ Men’s Negative attitude towards public Health services
✓ Availability of traditional healers	✓ Self-medication
✓ Availability of community forums	✓ Cultural practices in dealing with diseases
✓ Availability of Guidelines for good practice in the healthcare professions	✓ Masculinity beliefs
	✓ Use of traditional healing
	✓ Control over one’s own health
	✓ The stigma attached to health services utilisation
	✓ Peer influence

## Findings of the BOEM Analysis and Building Strategies

BOEM analysis revealed that there was a need to build on identified strengths, overcome identified weaknesses, explore opportunities, and minimise identified threats to address men’s poor health-seeking behaviour. The outcomes of BOEM are presented in Table 3.

**Table 3.** Results of BOEM Analysis and Building Strategies

Building on the strength	Overcome weaknesses
✓ Recognise the best employee of the month to motivate clinicians serving males.	✓ Introduce male-dedicated sections to prioritise men visiting public health facilities. This will help reduce waiting time at health facilities.
✓ Applaud professional nurses and other health care workers within the facility for patient satisfaction to encourage them to do more.	✓ Introduce a booking system for male patients coming for follow-up visits and give them a date and time to visit the facilities to see a clinician.
✓ Encourage men to utilise health services to encourage other men in the community to come to the health facilities and use services.	✓ Encourage clinicians to communicate reasons for delay to attend patients at health facilities, which will help reduce impatience while waiting.
✓ Educate men about services offered at public health facilities at no cost.	✓ Provide regular staff training on professional code of conduct and communication skills,
	✓ Recruit and employ more male nurses in health facilities
	✓ Recruit and employ more male Community health workers.

✓ Explore opportunities	✓ Minimising threats
✓ Use social media platforms such as Facebook, X (previously known as Twitter), and Podcasts to educate men about the importance of utilising health services and regular disease screening.	✓ Providing good quality services to men visiting public health facilities
✓ Introduce telemedicine to provide remote access to healthcare services.	✓ Change attitudes towards public health services
Work with traditional healers and encourage them to refer clients to health facilities for further management when they realise that their condition requires health services intervention.	✓ Provide community education
	✓ Work with traditional healers and address any misconceptions
	✓ Educate men about masculinity beliefs, how they impact their health, and why they should discard them.
	✓ Educate communities and the public about a variety of services offered at health facilities
	✓ provide in-service training for employers
	✓ Conduct a patient satisfaction survey to determine the patients’ satisfaction level.

### Proposed Strategies

In line with the study objective, a total of 14 strategies were developed and proposed to enhance men’s health-seeking behaviour. The strategies include implementing the best employee recognition programme for employees serving males in health facilities, intensifying compliance with policies and guidelines applicable to health service provision and ethical conduct on health workers servicing males, and implementing community outreach campaigns targeting males, which are presented in Table 4. The strategies were shared with stakeholders to review and give input. The stakeholders were experts in the field and community leaders.

**Table 4.** Proposed Strategies to Enhance Men’s Health-Seeking Behaviour in Limpopo Province, South Africa

Strategy	Purpose
1. Implementation of the best employee recognition programme for employees serving males in health facilities	✔ Encourage employees to engage with male patients respectfully and appropriately.
2. Implementation of male patients’ satisfaction surveys	✔ Determine male patients’ satisfaction level, and identify concerns and areas that need to be improved in the provision of health care services.
3. Intensify compliance with policies and guidelines applicable to health service provision and ethical conduct.	✔ Ensure compliance with policies and guidelines governing health services provision and ethical conduct of health professionals towards male patients.
4. Reduction of waiting period for males in health facilities	✔ Reduce the time male patients spend in the health facility.
5. Regular staff training	✔ Train health workers to communicate effectively with male patients
	✔ Encourage health workers to communicate the reasons and factors contributing to delays in providing patients with services in the health facility.
	✔ Remind health workers of guidelines and policies applicable in providing health services and interacting with patients, including males.
	✔ Encourage health workers to adhere to ethical guidelines
	✔ Ensure compliance with policies and guidelines governing health services provision and ethical conduct
6. Employ male nurses and community health workers (CWHs)	✔ To accommodate men who visit public health facilities and feel uncomfortable being assisted by female clinicians.
	✔ Use CWHs to encourage men to utilise health services and treatment adherence
7. Use available technology to influence men’s health-seeking behaviour	✔ Use social media platforms such as Facebook, X (previously known as Twitter), and Podcasts to educate men about the importance of utilising health services and regular disease screening.
	✔ Promote services offered at public health facilities tailored for men and the benefits of the services.
	✔ Create adverts that display the benefits of utilising health services to encourage men to utilise health services.
	✔ Provide remote access to health services using e-health platforms such as telemedicine.
8. Collaborate with community leaders, Faith-Based Organisations (FBOs), and Community-Based Organisations (CBOs)	✔ Create a working relationship with community leaders, Faith-Based Organisations (FBOs), and Community-Based Organisations (CBOs).
9. Collaborate with traditional healers	✔ Establish working relationships with traditional healers serving males
10. Implement community outreach campaigns targeting males	✔ Change men’s negative attitude towards public health services
	✔ Educate males about how masculine beliefs and control over one’s health affect individual health.
	✔ Educate the community, including men, about service packages offered in health facilities to reduce the stigma attached to using health services.
	✔ Cultural practices and traditional healing in dealing with diseases
11. Advocacy for employed males to access health services	✔ Advocate for employed males to be given time off and allowed to access health services.
12. Proper monitoring of the implementation of the South African National Integrated Men’s Health Strategy 2020-2025	✔ Ensure proper implementation of the South African National Integrated Men’s Health Strategy 2020-2025
13. Introduction of male-dedicated sections in existing health facilities	✔ Enable male patients who do not feel comfortable being assisted by female clinicians to be assisted by male clinicians.

## Discussion

The 14 developed strategies directly address identified behavioural, cultural and health system factors, thereby fulfilling the study’s objective of improving men’s health-seeking behaviour in Limpopo Province, South Africa. The strategies were validated by means of expert consultation, where a total of 12 experts were consulted. A total of 87 stakeholders also validated the strategies by critiquing and giving their opinions on the strategies. Inconsistent with SWOT analysis findings, the strategies suggest that effective monitoring and enforcement of policies, supported by regular training, may improve ethical conduct and improve service quality. Improved quality of service could motivate more men to use healthcare services. Ethics-based nursing practice has proven to positively impact healthcare practice.^16^ A study by Weske et al (2018)^17^ indicates that compliance is not just about enforcing rules, but also about the motivation to comply. However, in a situation where compliance with policies and guidelines of healthcare practice is not enforced, this strategy may not be effective in ensuring that the conduct of health workers encourages clients, including men, to use health services. The strategies highlight the role of community engagement and education in improving men’s health-seeking behaviour. When communities are engaged, they are more likely to have the right information, which could help clear myths and reduce the stigma associated with health service use. A study by Tshuma eta al. (2024)^18^ indicates that community members who participated in the community engagement dialogues prioritised men’s health-seeking behaviour and accessing health services. Haldane et al (2019)^19^ also state that community participation has positive impacts on the communities, individuals and improves health outcomes, and empowerment. Moreover, the strategies highlight structural and organisational challenges within the healthcare system that may affect the delivery of effective and quality healthcare services. The strategies suggest that improvements in healthcare system organisation and service delivery may enhance the quality and effectiveness of healthcare services provided to men. However, financial challenges that the South African public healthcare system faces may make it difficult to restructure and ensure optimal function of the public health system. Moreover, the system also experiences staff shortages, while more than 80% of the South African population relies on the public health system to access health services.^20^ While digital health platforms may improve access and engagement, they may also contribute to unintended consequences such as self-medication, suggesting the importance of appropriate oversight and professional guidance. Sawesi et al (2016)^21^ state that technology platforms enhance patient engagement in the healthcare process, improve quality of care, support healthcare safety, and provide cost-effective health services for patients. Despite making health services easily accessible, technology also negatively impacts health-seeking behaviour. For instance, a study conducted by Lin and Lin (2024)^22^ in China established that mobile Internet use promoted self-medication, and fewer adults used primary care facilities. The identified strategies also highlighted employment-related barriers that may affect men’s access to healthcare services. The findings suggest that collaboration between the Department of Employment and Labour, the Department of Health, and employers may support efforts to improve healthcare access for employed men. Existing workplace policies and labour-related practices may influence men’s ability to attend healthcare consultations, particularly during working hours. Previous evidence suggests that workplace support and advocacy initiatives may contribute to improved treatment retention, disease screening coverage, and healthcare utilisation patterns among employed men. However, giving men time off to attend health consultation sessions requires strict monitoring to prevent abuse of time off. It may also be difficult to achieve this strategy when employers are not willing to cooperate in improving employed men’s access to health services. The strategies also highlighted the role of patient feedback in identifying areas requiring improvement within healthcare services. Patient feedback may assist the health department in understanding service users’ experiences and identifying factors that influence satisfaction with healthcare services. These findings further suggest that collaboration with service users may support quality improvement initiatives within healthcare facilities. Moreover, communities in Limpopo are characterised by people who strongly believe in their culture, including how some health issues are dealt with. The Vatsonga, Vhavenda and Bapedi cultures are rooted in expectations of masculine strength, endurance, and not showing vulnerability. This could affect the effectiveness of strategies to promote men’s health-seeking behaviour and utilisation of public health services. Furthermore, traditional authorities were identified as influential stakeholders in the implementation of strategies aimed at addressing cultural influences on men’s health-seeking behaviour. The findings suggest that collaboration with traditional leaders may support community-level efforts to improve men’s utilisation of healthcare services. In African black communities, traditional leaders hold the highest authority and are respected; community members listen and obey them. It is easier for men to obey the rules of their leaders, and this could be taken as an advantage to encourage men to utilise health services. The use of traditional healing to address health issues is also common in this setting, and this could affect the effectiveness of the strategies on encouraging men’s utilisation of healthcare services. The strategies suggest that collaboration with traditional healers may support efforts to improve men’s utilisation of healthcare services. Moreover, culture also plays a vital role in communication through local languages, where respectful communication practices may influence the acceptability and effectiveness of the strategies within the setting. Moreover, the proposed strategies also align well with international approaches used to improve men’s health-seeking behaviour and engaging with health services, especially those aiming to promote access, improve provider attitude and influence gender norms. Similarly, male clinics have been used in Kenya to improve men’s health-seeking behaviours and have demonstrated the impact of gender norms on men’s use of health services.^10^ Moreover, evidence from Australia’s national men’s health policy emphasises how crucial male-friendly services are to improving men’s health-seeking behaviour. These services include flexible operating hours, clear communication, and supportive staff interactions. Community-based programmes like Australia’s “Men’s Sheds” highlight the importance of establishing informal, male-focused areas that break down social barriers and encourage participation through appropriate modes of communication.^8^ Evidence from other low- and middle-income countries further highlights the importance of addressing restrictive gender norms, enhancing healthcare worker training, and engaging men across the life course to improve service uptake.^23,24^The above approaches underscore the relevance of the current study’s proposed strategies for improving men’s health-seeking behaviours globally.

Implementation of these strategies requires the availability of funds, enough human resources, and time allocation. To ensure an effective electronic filing system, outreach campaigns, and hiring of male nurses and CHWs require additional budget. Due to budget constraints that the Department of Health experiences, it might be difficult to implement some of the recommended strategies, such as an electronic filing system and employing additional male nurses. The Department should consider expenses that come with the implementation of the strategies and allocate a budget to ensure successful implementation. Moreover, health facilities in rural settings of Limpopo often experience staff shortages, which may affect implementation of the strategies when there is no manpower to carry out required activities, such as conducting outreach campaigns. Furthermore, available staff may lack time to conduct dialogues and campaigns recommended by the strategies. Adequate staffing is required for the successful implementation of the strategies. Moreover, there are few employed male nurses in most rural health facilities, which is likely to affect the successful implementation of the strategies if additional male nurses are not employed. For instance, male dedicated sections may not be established if there are no male nurses to run the sections. Furthermore, the time frame to implement the strategies varies; some strategies require a longer period of implementation before they can be evaluated for effectiveness, while others require a shorter period. Patient satisfaction survey can be implemented quickly, while employment of male nurses and community workers and establishing male sections may take a longer period due to the process involved, including budgeting, recruitment and employing.

The developed strategies have implications for improving men’s health-seeking behaviour, given the number of actions suggested to encourage men to utilise healthcare services. For practice, the study recommends that the quality of care and patient experience should be improved by strengthening confidentiality practices and promoting respectful and non-judgmental communication by health professionals. Community engagement should be strengthened by collaborating with traditional leaders and offering health-seeking behaviour education and disease prevention and screening.

For policy, implementation monitoring of the South African National Integrated Men’s Health Strategy (2020–2025) should be improved. There is also a need to develop a policy that advocates for the recruitment of male healthcare providers. The strategies can help identify specific policy gaps in the health sector that contribute to men’s poor health-seeking behaviour. For research, an evaluation study could be conducted to measure how well these strategies are being implemented and what outcomes have been yielded. The study has potential limitations; it only focused on general males and could not include other male population groups, such as men who have sex with men. As a result, it developed strategies outlining actions to improve general males’ health-seeking behaviour, not other male population groups. Moreover, the study was conducted in Limpopo Province, which has its unique socio-cultural and health system challenges; as a result, the findings and proposed strategies may not be fully generalised to other provinces or different international settings. The quantitative phase employed a cross-sectional design, which limits the establishment of causal relationships between variables. In the qualitative phase, participants were purposively selected, and this limits the generalising findings beyond study participants. The study focused on developing strategies and could not implement them; therefore, the impact and feasibility remain unknown. Future studies should focus on implementing the suggested strategies to determine their effectiveness in improving men’s health-seeking behaviour. Furthermore, the study relied on self-reported data during the empirical phases. The phase 1(systematic review) cut-off date of 2020 possibly excluded studies reporting on new technology developments used to improve health-seeking behaviour. There is a possibility of publication overlap across the study phases.

## Conclusion

The findings of the conducted studies have justified the need to develop strategies to address men’s poor health-seeking behaviour. The findings justified the need for proposed adjustments and other actions within the healthcare sector and at a community level to improve men’s health-seeking behaviour. Internal issues within the healthcare sector, such as training, compliance, and quality of services, have been identified as some of the areas that need to be strengthened within the healthcare sector by the Department of Health to improve men’s health-seeking behaviour.

## Supplemental Material


Supplemental Material - Empowering Men’s Health: Strategies to Enhance Health-Seeking Behaviour in Rural Limpopo, South
Supplemental Material for Empowering Men’s Health: Strategies to Enhance Health-Seeking Behaviour in Rural Limpopo, South by Lazarros Chavalala, Lufuno Makhado and Rachel Tsakani Lebese in Health Services Insights.

Supplemental Material - Empowering Men’s Health: Strategies to Enhance Health-Seeking Behaviour in Rural Limpopo, South
Supplemental Material for Empowering Men’s Health: Strategies to Enhance Health-Seeking Behaviour in Rural Limpopo, South by Lazarros Chavalala, Lufuno Makhado and Rachel Tsakani Lebese in Health Services Insights.

Supplemental Material - Empowering Men’s Health: Strategies to Enhance Health-Seeking Behaviour in Rural Limpopo, South
Supplemental Material for Empowering Men’s Health: Strategies to Enhance Health-Seeking Behaviour in Rural Limpopo, South by Lazarros Chavalala, Lufuno Makhado and Rachel Tsakani Lebese in Health Services Insights.

## Data Availability

Data is available from the corresponding author on formal written request.*
