# Supplementary material for: Empowering Men’s Health: Strategies to Enhance Health-Seeking Behaviour in Rural Limpopo, South Africa
Source: Health Serv Insights. 2026 Jul 31;19:11786329261472590. doi: 10.1177/11786329261472590 (PMC13428122; doi:10.1177/11786329261472590)
Supplement: Supplemental Material - Empowering Men’s Health: Strategies to Enhance Health-Seeking Behaviour in Rural Limpopo, South [file sj-pdf-1-his-10.1177_11786329261472590.pdf]

Good Reporting of A Mixed Methods Study (GRAMMS) checklist

Guideline	Section: page
Describe the justification for using a mixed methods approach to the research question	Methods, page 3
Describe the design in terms of the purpose, priority and sequence of methods	Methods- page 3
Describe each method in terms of sampling, data collection and analysis	Methods, page 3-8
Describe where integration has occurred, how it has occurred and who has participated in it	Methods, page 8-9
Describe any limitation of one method associated with the present of the other method	Discussion page 18
Describe any insights gained from mixing or integrating methods	Discussion: page 14-17

O'Cathain A, Murphy E, Nicholl J. The quality of mixed methods studies in health services research. J Health Serv Res Policy. 2008;13: 92-98.
