# Supplementary material for: Empowering Men’s Health: Strategies to Enhance Health-Seeking Behaviour in Rural Limpopo, South Africa
Source: Health Serv Insights. 2026 Jul 31;19:11786329261472590. doi: 10.1177/11786329261472590 (PMC13428122; doi:10.1177/11786329261472590)
Supplement: Supplemental Material - Empowering Men’s Health: Strategies to Enhance Health-Seeking Behaviour in Rural Limpopo, South [file sj-pdf-2-his-10.1177_11786329261472590.pdf]

Developed strategies' actions and indicators

Strategy	Action	Indicators
**1. Implementation of the best employee recognition programme for employees serving males in health facilities**	✔ Facility managers and section managers should identify employees who performed well ,,Supplementaryand give the best employees certificates of recognition for good performance every month.<br>✔ Recognition programme can target health workers working directly with patients, such as nurses and admin staff.	✔ Number of employees who were recognised
**2. Implementation of male patients' satisfaction surveys**	✔ Give each male patient an anonymous satisfaction survey form to complete after the treatment session and analyse the data for service satisfaction insights.	✔ Number of male patients who completed satisfaction survey forms.
**3. Intensify compliance with policies and guidelines applicable to health service provision and ethical conduct.**	✔ Take disciplinary actions against health workers for ethical misconduct	✔ Number of health workers disciplined for ethical misconduct
**4. Reduction of waiting period for males in health facilities**	✔ Implement an electronic filing system that allows healthcare workers to quickly access patient records, eliminating the need to keep patients waiting while retrieving hard copies from shelves.<br>✔ Allocate slots for male patients and stick to booked appointments<br>✔ Introduce an appointment management system to manage the number of patients booked for the day and reserve appointments for emergencies.<br>✔ Stick to appointments when providing services except for emergency cases	✔ Average time each male patient spent in the facility
**5. Regular staff training**	✔ Provide staff development training on healthcare professionals' ethics, policies guiding health services provision, and conduct communication skills training for working with male clients.	✔ Number of healthcare workers trained on communication skills, ethics and policies guiding health services provision
**6. Employ male nurses and community health workers (CWHs)**	✔ Sub-district and District managers of the Department of Health need to write motivational letters to the Provincial Department of Health for the recruitment of male nurses and male community health workers.	✔ Number of male nurses and male CHWs employed
**7. Use available technology to influence men's health-seeking behaviour**	✔ Create or use available Sub-District, District, and Provincial Departments of Health Facebook, X, and podcasts to educate men about the importance of using health services, using local languages to make it easy for men to understand the message.<br>✔ Introduce free-to-use mHealth platforms that can be accessed through cell phones, tablets and computers to enable men to interact with a doctor or nurse without physically meeting with the healthcare professional.<br>✔ Introduce free-to-use mobile apps that can be downloaded and installed on a mobile phone and share health education messages on men's health every week.<br>✔ Create appointment systems that will allow patients to book appointments and enable the facility to manage booked appointments.<br>✔ Create an appointment reminder system that will send reminders to patients about pending booked appointments.	✔ Several men reached out through social media platforms.<br>✔ Several adverts displaying the benefits of using health services were created.<br>✔ Number of men accessing health services using e-health

**8. Collaborate with community leaders, Faith-Based Organisations (FBOs), and Community-Based Organisations (CBOs)**	✔ Facility managers make requests to meet community leaders and faith-based and community-based organisations.<br>✔ During the meeting, inform the stakeholders of the health-seeking behaviour problem among men and request stakeholders to work with the health sector to improve men's health-seeking behaviour in their communities. Encourage community leaders and faith-based and community-based organisations to encourage their male members to utilise health services.<br>✔ On an ongoing quarterly basis, facility managers meet with community leaders, faith-based organisations and community-based organisations to provide updates on men's health-seeking behaviour. The health facilities will also share observations on progress made by men in health services utilisation.	✔ Number of stakeholders collaborating
**9. Collaborate with traditional healers**	✔ Facility managers make requests to meet local traditional healers<br>✔ During meetings, nurses share knowledge with traditional healers on the importance of consulting at health facilities.<br>✔ Nurses encourage traditional healers to refer their male clients to health facilities for consultation to identify conditions that they may not be able to diagnose from their perspective.	✔ A number of traditional healers collaborate.<br>✔ Number of males referred to health facilities
**10. Implement community outreach campaigns targeting males**	✔ Professional nurses to request community leaders, faith-based and community-based organisations to mobilise their members for campaigns.<br>✔ During campaigns, educate members about health services offered in public health facilities, the impact of masculinity beliefs on health-seeking behaviour and inform members that using health services does not take away control over their health.	✔ A number of males reached through community outreach campaigns.
**11. Advocacy for employed males to access health services**	✔ The Department of Health, through the Department of Labour, requests to provide in-service training to employers about the importance of allowing employees to utilise health services.<br>✔ Encourage employers to adhere to the Basic Conditions of Employment Act and other relevant legislation to monitor employees' absenteeism due to attendance at health services to minimise abuse of sick leaves.	✔ A number of employed males accessing health services.
**12. Proper monitoring of the implementation of the South African National Integrated Men's Health Strategy 2020-2025**	✔ Follow up on health facilities to establish if proposed activities in the strategy are carried out as per plan.	✔ Number of males offered services in line with the South African National Integrated Men's Health Strategy 2020-2025.
**13. Introduction of male-dedicated sections in existing health facilities**	✔ The Department of Health should recruit male nurses according to their categories in each health facility.<br>✔ The Department of Health should create male-dedicated sections and allocate male nurses to provide services to males within health facilities.	✔ A number of health facilities with male-dedicated sections.<br>✔ A number of male patients offered services in male-dedicated sections.
