# Supplementary material for: Empowering Men’s Health: Strategies to Enhance Health-Seeking Behaviour in Rural Limpopo, South Africa
Source: Health Serv Insights. 2026 Jul 31;19:11786329261472590. doi: 10.1177/11786329261472590 (PMC13428122; doi:10.1177/11786329261472590)
Supplement: Supplemental Material - Empowering Men’s Health: Strategies to Enhance Health-Seeking Behaviour in Rural Limpopo, South [file sj-pdf-3-his-10.1177_11786329261472590.pdf]

Paper 1 Findings	Paper 2 findings	Paper 3 findings	Paper 4 findings	Paper 5 findings	Paper 6 findings	Merged analysis of findings
**Strategies used to promote health-seeking behaviour**<br>✓ Peer education<br>✓ Men's sheds<br>✓ Health self-testing<br>✓ Telemedicine and digital platforms<br>✓ Health education programmes<br>✓ Male clinics	✓ **Men's health awareness, sources of information and perceived health status**<br>✓ Men held average knowledge about their health<br>✓ Healthcare workers and the internet were sources of health information<br>✓ Men perceived their health status as being good.	**Attitude of men towards public healthcare services and utilisation**<br>✓ Men held negative attitudes towards public health services<br>✓ Men use public healthcare services irregularly	**Men's views on factors contributing to their poor health-seeking behaviour**<br>✓ Self-medication<br>✓ Fear of health status and disease screening<br>✓ Use of traditional healing services and belief in traditional meds<br>✓ Peer, family and community influence<br>✓ Health services use Stigma Cultural beliefs	**Men's experiences of health services at visited public health facilities in Limpopo Province, South Africa**<br>✓ Poor service<br>✓ Confidentiality breach<br>✓ Felt uncomfortable with female nurses<br>✓ Gender discrimination<br>✓ Verbal abuse Clinical Unethical Conduct<br>✓ Polite advice and patient education<br>✓ Difficult to open for female nurses	**Professional Nurses' Views on factors contributing to poor health seeking among men: experiences of providing male patient services**<br>✓ Cultural factors,<br>✓ fear of knowing one's own health status,<br>✓ The stigma attached to visiting health facilities, peer influence, and employment<br>✓ Socio-economic factors<br>✓ Consult when the disease is in a severe state<br>✓ Men lie about symptoms of illness<br>✓ Men are always in a hurry and expect the session to end quickly<br>✓ Appreciate respect<br>✓ Refuse a private parts physical examination by female clinicians	✓ There is a need to consider Behavioural,<br>✓ Cultural and Health system factors when developing intervention strategies<br>✓ Most men had bad experiences in public health facilities when seeking health careSupplementary<br>✓ Previously implemented strategies<br>✓ Addressed some of the identified factors; however, some gaps led strategies to be ineffective.<br>✓ There is a need to strengthen and expand healthcare workers' information-sharing platforms with men.<br>✓ Patient satisfaction should be strengthened to encourage health service use.
